# Discordance between self-reported arthritis and musculoskeletal signs and symptoms in older women

**DOI:** 10.1186/s12891-016-1349-4

**Published:** 2016-12-01

**Authors:** TKT Lo, Lynne Parkinson, Michelle Cunich, Julie Byles

**Affiliations:** 1 Research Centre for Gender, Health and Ageing, HMRI, University of Newcastle, C/- University Drive, Callaghan, NSW 2308 Australia; 2 Central Queensland University, Rockhampton, QLD 4701 Australia; 3 Faculty of Pharmacy, Charles Perkins Centre, The University of Sydney, Camperdown, NSW 2006 Australia; 4 School of Medicine and Public Health, The University of Newcastle, Callaghan, NSW 2308 Australia

**Keywords:** Arthritis, Geriatrics, Health survey, Self-report, Validity, Women

## Abstract

**Background:**

Arthritis is a gendered disease where women have a higher prevalence and more disability than men with arthritis of the same age. Health survey data is a major source of information for monitoring of the burden of arthritis. The validity of self-reported arthritis and the determinants of its accuracy among women have not been thoroughly studied. The objectives of this study were to: 1) examine the agreement between self-report diagnosed arthritis and musculoskeletal signs and symptoms in community-living older women; 2) estimate the sensitivity, specificity, and predictive values of self-reported arthritis; and 3) assess the factors associated with the disagreement.

**Methods:**

A cross-sectional survey of women was undertaken in 2012–13. The health survey asked women about diagnosed arthritis and musculoskeletal signs and symptoms. Agreement between self-reported arthritis and musculoskeletal signs symptoms was measured by Cohen’s kappa. Sensitivity, specificity, and predictive values of self-reported arthritis were estimated using musculoskeletal signs and symptoms as the reference standard. Factors associated with disagreement between self-reported arthritis and the reference standard were examined using multiple logistic regression.

**Results:**

There were 223 participants self-reported arthritis and 347 did not. A greater number of participants who self-reported arthritis were obese compared to those who did not report arthritis. Those who reported arthritis had worse health, physical functioning, and arthritis symptom measures. Among the 570 participants, 198 had musculoskeletal signs and symptoms suggesting arthritis (the reference standard). Agreement between self-reported arthritis and the reference standard was moderate (kappa = 0.41). Sensitivity, specificity, and positive and negative predictive values of self-reported arthritis in older women were 66.7, 75.5, 59.2, and 81.0% respectively. Regression analysis results indicated that false-positive is associated with better health measured by the Short Form 36 physical summary score, the Health Assessment Questionnaire disability index, or the Western Ontario and McMaster University Osteoarthritis Index total score; whereas false-negative is negatively associated with these variables.

**Conclusion:**

While some women who reported diagnosed arthritis did not have recent musculoskeletal signs or symptoms, others with the signs and symptoms did not report diagnosed arthritis. Researchers should use caution when employing self-reported arthritis as the case-definition in epidemiological studies.

**Electronic supplementary material:**

The online version of this article (doi:10.1186/s12891-016-1349-4) contains supplementary material, which is available to authorized users.

## Background

Arthritis is very common and a leading cause of pain and disability around the world [1–3]. A considerable amount of healthcare resources is dedicated by the governments to the management of arthritis [1, 4–6]. It is estimated that over 50 million people are living with arthritis in the USA [4], while over 10 million and three million people are affected by the disease in the UK and Australia respectively [1, 7]. Arthritis is also a gendered disease, where women are more likely to be affected than men [1, 3–5, 8]. For example, osteoarthritis (i.e. the most common form of arthritis) affects women more severely and at more sites [8–10]. Consequently, women with arthritis account for more healthcare utilisation than do men with arthritis at the same age [6, 9, 11]. The rate of joint replacements (knee replacements particularly) performed in women is also much higher than that for men, reflecting both the higher prevalence and the worse severity of arthritis in women [9, 11]. As managing arthritis poses a considerable challenge to the limited resources in the healthcare systems and affects the quality of life of millions of women, it is important to monitor the burden of arthritis.

Self-report heath survey data is a major source of information for epidemiological studies and other health research [12, 13]. Use of self-reported health data is feasible because health survey data are often routinely collected by government departments and/or agencies (especially in developed countries) and are readily available and accessible [12–14]. Self-reported diagnosed arthritis is among the most commonly used case-definition for prevalence and other epidemiological studies of arthritis burden [15–25]. Although it has been argued that self-reported diagnosis of chronic conditions may suffer from recall-bias, which could lead to *underreporting* of conditions and underestimation of prevalence [15, 26–28], some researchers have justified the use of self-reported arthritis as it has good agreement with medical records [18–20], and an adequate level of sensitivity and specificity in previous validation studies [22]. However, it is acknowledged that generalization of the findings of validation studies from one population to another may be inappropriate due to the differences in socio-demographic, lifestyle, and health characteristics which may affect the willingness of individuals to report medical conditions and/or seek healthcare [21–25]. For example, there is evidence indicating women tend to *overreport* (i.e. instead of underreport) arthritis in health survey compared to men [29]. Nonetheless, previous validation studies of self-reported arthritis have mostly been based on a non-gender specific sample and/or have not performed stratified analysis by gender [30–33]. Since women are most at risk of arthritis, a study with a particular focus on women represents an important step to the better understanding of the validity of self-reported arthritis and its application in large epidemiological studies.

The objective of this study is to examine the accuracy of self-reported arthritis as the case-definition in community-living women for the epidemiological study of arthritis. The specific aims are threefold: 1) to assess the agreement between self-reported diagnosed arthritis and musculoskeletal signs and symptoms suggesting arthritis in older women; 2) to assess the accuracy of self-reported arthritis based on the sensitivity, specificity, and predictive values using musculoskeletal signs and symptoms as the reference; and 3) to examine the factors associated with disagreement between self-reported arthritis and musculoskeletal signs and symptoms.

## Methods

### Participants

The Australian Longitudinal Study on Women’s Health (ALSWH) is a population-based survey of women that began in 1996 [34]. ALSWH participants were randomly selected from the national health insurance database [35]; they broadly represented the women in Australia at that time [36]. ALSWH is designed to investigate multiple factors that affect the health and well-being of women [35]. Since arthritis is a gendered disease and women are particularly at risk [1, 3–6, 8, 9], ALSWH provided an appropriate sampling frame for this study.

### Data collection

A cross-sectional survey of a sample of women from the 1946–51 birth cohort of ALSWH was undertaken between December 2012 and March 2013. Postal self-administered questionnaires were sent to 350 randomly selected women who previously self-reported arthritis in Survey 3 (2001) and/or Survey 4 (2004), and another 350 women who had never reported arthritis in the ASLWH. Reminder leaflets were sent to non-respondents 30 days after the initial mail-out. Details of the protocol for this health survey have been published [37].

### Self-reported diagnosed arthritis

In the survey questionnaire, participants were asked: “In the past 3 years, have you been diagnosed or treated for (a list of conditions)?” The forms of arthritis listed were osteoarthritis, rheumatoid arthritis, psoriatic arthritis, gout, and/or other form of arthritis. Self-reported diagnosed arthritis in the present study was defined as an answer of “Yes” to any form of arthritis.

### Reference standard for arthritis

The reference standard definition of arthritis was based on the reported musculoskeletal signs and symptoms suggesting arthritis. A set of musculoskeletal signs and symptom questions were adapted from the Community Oriented Program for Control of Rheumatic Disease (COPCORD) Core Questionnaire (CCQ) [38]. The COPCORD-CCQ was originally designed by the WHO and the International League against Rheumatism (ILAR) as a screening tool for rheumatic symptoms and disabilities in the community [39]. COPCORD-CCQ has been applied to study the prevalence of rheumatic diseases among community-living individuals in Australia, [40] and other countries [38]. The CCQ has established high validity as rheumatic disease screening and diagnostic tools [41, 42]. Simplified versions of COPCORD type questionnaires have been proposed [42, 43], and four variables (i.e. pain in the last 7 days, high pain score, a Health Assessment Questionnaire score of greater than 0.80, and previous diagnosis) have been shown to perform well in the identification of osteoarthritis and rheumatoid arthritis cases in the community [43]. While another study has demonstrated that two questions: 1) “In the last 7 days (or ever) have you had any problem, that is pain, tenderness (pain on pressure), swelling or stiffness in your bones, joints and muscles?” and responding “Yes”; and 2) “Was there a traumatic event (such as strain or injury) that caused the pain, tenderness, swelling or stiffness?” and responding “No”; can be used to detect rheumatic disorders such as arthritis in the general population [42]. Thus, the reference case-definition of arthritis in the present study was defined as: 1) reported pain, tenderness, swelling or stiffness in bones, joints or muscles in the last week; 2) that this pain was not caused by a traumatic event; and 3) that the pain was at least mild in severity (i.e. level three or greater on the 0–10 scale). Confirmation of diagnosis based on signs and symptoms that include joint pain, tenderness, swelling, stiffness and reduced mobility also aligns with the recommendations made by the Royal Australian College of General Practitioners (RACGP) about the clinical examination of osteoarthritis and rheumatoid arthritis [44, 45].

### Socio-demographic, lifestyle, and health variables

To describe characteristics of the sample, socio-demographic, lifestyle, and health variables were used. Inclusion of these variables was guided by the literature, where variables that were shown to be associated with arthritis or false-reporting of arthritis were included [22, 29–31, 33, 46]. The socio-demographic variables included were age, marital status, area of residence, and level of education. The lifestyle variables were current smoking status and obesity. Obesity was defined as a body mass index (BMI) equal to or greater than 30, i.e. according to the WHO criteria [47]. The health variables were the Short Form 36 (SF-36) quality of life measures [48], the Health Assessment Questionnaire (HAQ) disability index [49], the Western Ontario and McMaster Universities Osteoarthritis Index (WOMAC) [50], and a list of chronic conditions that are common in older women. The SF-36 measures included the physical (PCS) and mental (MCS) component summary scores, which range from 0 to 100, and higher scores represent better health [48]. The HAQ disability index assesses functional ability in eight categories including dressing, rising, eating, walking, hygiene, reach, grip, and usual activities [49]. The WOMAC was developed to measure symptoms and physical disability for individuals with osteoarthritis of the hip and knee, and evaluates pain, stiffness, and physical functions, where higher score represents worse symptoms [50]. The list of chronic conditions included anxiety, asthma, bronchitis/emphysema, depression, diabetes, heart disease, hypertension, low iron levels, osteoporosis, and thrombosis.

### Statistical analysis

Characteristics of the participants who self-reported diagnosed arthritis and those who did not report arthritis in the survey questionnaire were compared using t-tests (for normally distributed continuous variables), Wilcoxon Mann–Whitney tests (for non-normally distributed continuous variables), and chi-square tests (for categorical variables) [51]. A priori two-tailed α level of 0.05 was used for all statistical tests.

Agreement between self-reported diagnosed arthritis and the reference standard was measured by Cohen’s kappa (κ), which is a chance adjusted measure of agreement [52]. Sensitivity, specificity, and the predictive values of self-reported arthritis were also estimated. For this study, a true-positive was defined as a case identified by both self-reported arthritis and musculoskeletal signs and symptoms, whereas a false-positive was defined as a case identified by self-reported arthritis but not ascertained by the reference standard.

Logistic regression was used to assess the characteristics of women associated with disagreement between self-reported arthritis and the reference standard, with separate models for: 1) false-positives; and 2) false-negatives. The lifestyle and health variables were of particular relevance. Overweight and obesity have been linked to arthritis [8], and individuals who are obese has been shown to be associated with overreporting of arthritis [31]. Health variables including PCS and the number of activities of daily living (ADL) limitations have been linked to false-reporting of arthritis [30, 31]. However, the directions of association have not been consistent. For example, in one report, self-related health was found to be *negatively* associated with overreporting of arthritis [31]; whereas in another report, physical health was found to be *positively* associated with overreporting [30]. In another study, *better physical health* was identified as a factor also positively associated with underreporting while ADL limitations (i.e. can be linked to *worse physical health*) was found to be a significant factor of underreporting [30]. In the current study, both SF-36 PCS and MCS measures, the HAQ, WOMAC total score, and comorbidity were included in the analysis. Comorbidity was a count of chronic conditions listed above [30]. However, health measures may be strongly correlated to each other. To avoid multicollinearity in the multivariable regression models, correlations of the health variables were assessed in the preliminary analysis (see below).

Potential explanatory variables for false-positive and false-negative were first examined using univariate analyses. Then, multivariable regression analyses were used to examine: a) the effects of the lifestyle variables after controlling for the socio-demographics, and b) the effect of health variables after controlling for both socio-demographic and lifestyle variables. Preliminary analysis indicated that the SF-36 PCS, HAQ, and WOMAC are strongly correlated (correlation coefficients >0.75). Hence, four multivariable regression models (a-d) were constructed for false-positive and false-negative. The models were those included: a) the socio-demographic and lifestyle variables only; b) the socio-demographics, lifestyle variables, comorbidity, and the SF-36 summary scores; c) the socio-demographics, lifestyle variables, comorbidity, and HAQ; and d) the socio-demographics, lifestyle variables, comorbidity, and the WOMAC score. Statistical analyses were performed in Stata IC version 11 (StataCorp LP, College Station, TX, USA).

## Results

**Fig. 1:**
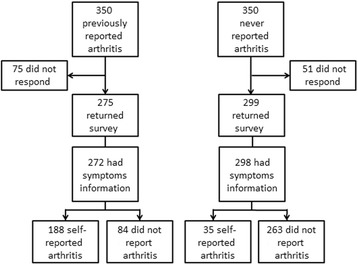
Flowchart of recruitment of study participants and their reported musculoskeletal symptoms and arthritis status. (*MSK* musculoskeletal)

As at 22nd March 2013, 574 women had completed and returned the survey questionnaire; the response rate was 82.0%. Among them, 570 women answered the sign and symptom questions. Analysis was based on data from these 570 women (i.e. 81.4% of the 700 women originally approached). The flow from the recruitment stage to the classification of women is illustrated in Fig. 1. Overall, women with and without self-reported diagnosed arthritis are not significantly different in socio-demographic characteristics; but an increased proportion were obese, had more chronic conditions, worse quality of life (PCS and MCS), greater disability, and worse in the WOMAC total score compared to women without arthritis. Listed in Table 1 are the characteristics of the sample.

**Table 1:** Demographic and health characteristics of study participants

Characteristics	Without self-report arthritis *n* = 347	With self-report arthritis *n* = 223
Age (mean [SD], year)	63.6 [1.5]	63.6 [1.4]
Married or de facto (%)	82.1	78.0
Residing in urban area (%)	40.2	34.3
High school level less (%)	52.9	56.9
Current smoker (%)	7.8	7.7
Obese (%)	59.7	73.5**
Comorbid conditions		
Anxiety (%)	6.92	9.87
Asthma (%)	9.22	16.59**
Bronchitis/emphysema (%)	4.9	6.28
Depression (%)	8.36	18.83**
Diabetes (%)	5.76	10.76*
Heart disease (%)	4.32	8.97*
Hypertension (%)	19.31	32.74**
Low iron levels (%)	4.61	4.93
Osteoporosis (%)	8.07	13.45*
Thrombosis (%)	0.29	1.35
PCS†, SF-36 (mean [SD])	48.6 [9.3]	39.4 [10.9]**
MCS†, SF-36 (mean [SD])	53.1 [8.2]	50.5 [11.0]**
HAQ Disability Index‡ (mean [SD])	0.2 [0.4]	0.6 [0.6]**
WOMAC total score‡ (mean [SD])	15.3 [14.5]	27.6 [18.7]**

*P ≤ 0.05, **P ≤ 0.01. *Abbreviations*: *SD* standard deviation, *SF-36* Short Form-36, *PCS* physical summary score, *MCS* mental summary score, *HAQ* Health Assessment Questionnaire, *WOMAC* Western Ontario and McMaster Universities Osteoarthritis Index

†Higher PCS and MCS scores indicate better health. ‡Higher HAQ and WOMAC scores indicate worse symptoms

**Fig. 2:**
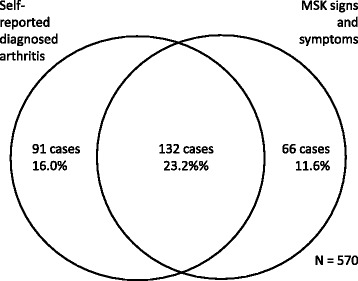
An illustration of the number of cases identified by the two definitions among older women. Self-reported diagnosed arthritis identified 223 (=91 + 132) cases and 198 (=132 + 66) cases were confirmed using on musculoskeletal (MSK) signs and symptoms. There were 132 common cases identified by both definitions

Crude prevalence of arthritis estimates based on self-reported arthritis and musculoskeletal signs and symptoms were not statistically significantly different; they were 39.1% (95% CI 35.1–43.3%) and 34.7% (95% CI 30.8–38.8%) respectively. The number of cases identified uniquely by either of the definitions and both case-definitions concurrently is depicted in the Venn diagram (Fig. 2). Agreement between self-reported arthritis and musculoskeletal symptoms was moderate (κ = 0.41, 95% CI 0.33–0.49). Sensitivity of self-reported arthritis was 66.7% (95% CI 60.0–73.3%), whereas specificity was 75.5% (95% CI 71.1–79.9%). Positive predictive value (PPV) and negative predictive values (NPV) of self-reported arthritis were 59.2% (95% CI 52.7–65.7%) and 81.0% (95% CI 76.8–85.1%) respectively. The contingency table is exhibited in Table 2.

**Table 2:** Self-reported arthritis versus musculoskeletal signs symptoms (*n* = 570)

		MSK signs & symptoms	
		with arthritis	without
Self-reported	with arthritis	132	91
	without	66	281

*MSK* musculoskeletal

When using musculoskeletal signs and symptoms as the reference standard, univariate analysis results indicated that false-positiveness of self-reported arthritis was associated with a higher level of education, being obese, or better physical health; and negatively associated with disability or a higher WOMAC score. They also indicated that false-negatives were associated obesity, disability or the WOMAC score; but negatively associated with physical health. Details of the univariate analysis results can be found in an additional file [see Additional file 1: Table S1].

Results of the multiple logistic regression models are illustrated in Tables 3 and 4. In Table 3, results show that after controlling for the socio-demographics, lifestyle variables including obesity were not significant predictors of false-positive (Model 1a). When controlling for both socio-demographic and lifestyle variables, better physical health (*P* < 0.001), a lower disability measure (*P* = 0.001) or WOMAC score (*P* = 0.003) is significantly associated with false-positive (Models 1b – 1d). Obesity is significantly associated with false-negative (*P* = 0.045) after controlling for socio-demographics (Model 2a in Table 4). However, when the health variables were simultaneously entered into the models, only comorbidity (*P* = 0.037) and physical health (*P* < 0.001, Model 2b), or greater disability (*P* < 0.001, Model 2c), or greater WOMAC scores (*P* = 0.001, Model 2d) were associated with false-negative.

**Table 3:** Multiple logistic regression models for factors associated with false-positive (*n* = 223)

Characteristics	False-positive							
	Model 1a		Model 1b		Model 1c		Model 1d	
	Odds ratio	(95% CI)	Odds ratio	(95% CI)	Odds ratio	(95% CI)	Odds ratio	(95% CI)
Age	0.97	(0.79–1.19)	0.98	(0.78–1.23)	1.01	(0.81–1.25)	1.00	(0.78–1.27)
Married or de facto	1.34	(0.66–2.73)	0.89	(0.39–2.05)	1.09	(0.51–2.31)	1.06	(0.47–2.40)
Residing in urban area	1.44	(0.80–2.61)	1.15	(0.60–2.22)	1.39	(0.75–2.57)	0.90	(0.45–1.83)
High school or less	0.76	(0.43–1.34)	0.79	(0.42–1.47)	0.93	(0.51–1.70)	0.80	(0.41–1.56)
Current smoker	1.85	(0.66–5.21)	1.56	(0.49–4.91)	1.92	(0.65–5.68)	1.65	(0.50–5.48)
Obese	0.68	(0.36–1.27)	0.63	(0.31–1.28)	0.77	(0.40–1.49)	0.74	(0.34–1.59)
Comorbidity			1.24	(0.95–1.62)	1.19	(0.94–1.51)	1.24	(0.96–1.61)
PCS†			1.08	(1.04–1.12)**				
MCS†			1.01	(0.98–1.05)				
HAQ Disability Index‡					0.33	(0.17–0.63)**		
WOMAC total score‡							0.97	(0.95–0.99)**

*P ≤ 0.05, **P ≤ 0.01. *Abbreviations*: *CI* confidence interval, *PCS* SF-36 physical summary score, *MCS* SF-36 mental summary score, *HAQ* Health Assessment Questionnaire, *WOMAC* Western Ontario and McMaster Universities Osteoarthritis Index

†Higher PCS and MCS scores indicate better health. ‡Higher HAQ and WOMAC scores indicate worse symptoms

**Table 4:** Multiple logistic regression models for factors associated with false-negative (*n* = 347)

Characteristics	False-negative							
	Model 2a		Model 2b		Model 2c		Model 2d	
	Odds ratio	(95% CI)	Odds ratio	(95% CI)	Odds ratio	(95% CI)	Odds ratio	(95% CI)
Age	1.08	(0.89–1.31)	1.02	(0.82–1.26)	1.07	(0.87–1.31)	1.11	(0.82–1.51)
Married or de facto	0.73	(0.36–1.50)	0.92	(0.40–2.10)	0.74	(0.35–1.56)	0.51	(0.16–1.56)
Residing in urban area	0.54	(0.29–0.99)*	0.59	(0.30–1.17)	0.60	(0.31–1.13)	0.49	(0.18–1.32)
High school or less	0.60	(0.34–1.06)	0.47	(0.25–0.88)*	0.56	(0.31–1.03)	0.74	(0.32–1.74)
Current smoker	0.91	(0.32–2.58)	0.99	(0.31–3.22)	0.96	(0.33–2.84)	1.20	(0.24–5.94)
Obese	1.85	(1.01–3.36)*	1.56	(0.79–3.09)	1.65	(0.87–3.14)	1.09	(0.42–2.86)
Comorbidity			0.68	(0.47–0.98)*	0.83	(0.61–1.13)	0.87	(0.57–1.32)
PCS†			0.92	(0.89–0.95)**				
MCS†			0.97	(0.93–1.01)				
HAQ Disability Index‡					5.09	(2.29–11.30)**		
WOMAC total score‡							1.06	(1.02–1.09)**

*P ≤ 0.05, **P ≤ 0.01. *Abbreviations*: *CI* confidence interval, *PCS* SF-36 physical summary score, *MCS* SF-36 mental summary score, *HAQ* Health Assessment Questionnaire, *WOMAC* Western Ontario and McMaster Universities Osteoarthritis Index

†Higher PCS and MCS scores indicate better health. ‡Higher HAQ and WOMAC scores indicate worse symptoms

## Discussion

This study compared self-reported diagnosed arthritis and musculoskeletal signs and symptoms suggesting arthritis in a sample of geographically diverse older Australian women. Prevalence estimates based on the two case-definitions of arthritis were not statistically significantly different, but Cohen’s kappa shows that their agreement was only moderate. While two-fifths (91/223) of the self-reported arthritis cases did not have musculoskeletal signs and symptoms, two-thirds (132/198) of cases identified by signs and symptoms also reported diagnosed arthritis. Although it has been suggested that women are more likely to consult a doctor for their conditions and lead to an increased chance of diagnoses [22], our results indicate there were some women in our sample who had joint signs and symptoms (suggesting arthritis) have not had a diagnosis of arthritis. Possible contributing factors to this finding may include cultural beliefs and awareness of arthritis. Some individuals may have believed joint signs and symptoms are an inevitable result of ageing and hence did not seek help from their doctor [10], despite the fact that the effects of arthritis can be reduced through early treatment and appropriate management.

Our results also indicate that self-reported diagnosed arthritis has moderate sensitivity and specificity when using musculoskeletal signs and symptoms as the reference standard. These results are somewhat different to those in previous studies that included both women and men [31, 53]. One USA study involving an older sample from Georgia and using rheumatologists’ summary assessment as the reference standard, found that self-reported arthritis had substantial agreement with the reference standard [31]. Another study with a similar methodology found that self-reported arthritis had high sensitivity and moderate specificity in a population aged 65 or older living in Massachusetts [53]. Aside from the sample differences between the studies, the agreement and other performance measures in the current study might have been underestimated due to our adoption of the COPCORD questions. We did not use rheumatologist’s diagnosis because we do not think it is an appropriate case-definition of arthritis in the community. In Australia, general practitioners are the first point of contact for people with arthritis [1]. The Royal Australian College of General Practitioners recommends that confirmation of a diagnosis of osteoarthritis (i.e. far more common than any other form of arthritis) should be based on clinical presentations such as joint pain, swelling, stiffness and reduced mobility [44]. This is consistent with our first question, which assessed musculoskeletal signs and symptoms that include pain, tenderness, swelling or stiffness in the joints, bones, and muscles. However, our adoption of the COPCORD-CCQ also means we assessed only the signs and symptoms “*in the last week*” [42]. This is where it is incompatible with the Royal Australian College of General Practitioners guidelines. In the guideline for the general practitioners, there is a lack of specification of the timeframe of the signs and symptoms being assessed [44].

The timeframe in the COPCORD questions is similar to that in the American College of Rheumatology (ACR) criteria for diagnosing osteoarthritis of the hip or knee, which examine case-ness based on joint pain experienced “on most days of the past month” [54, 55]. Both the COPCORD-CCQ and the ACR criteria require regular and frequent musculoskeletal symptoms for diagnosis. Yet, this definition may have been too restrictive for monitoring the burden of arthritis in community-living individuals [32]. It has been suggested that case-definitions that require frequent musculoskeletal symptoms can omit cases that have fluctuating symptoms, or whose symptoms (and/or signs) are controlled by regular medication [32]. Instead, questions about signs and symptoms “in the previous 6 months” [32], and/or a question about the use of arthritis-related medicines [29, 53], may better detect arthritis in the community. If we had used the above questions, then the reference standard would have included more cases, and the number of true-positives would have been higher and the number of false-negatives would have been lower. This would have a positive impact on the estimated agreement and the four performance measures (i.e. sensitivity, specificity, PPV, and NPV) in our study.

Our results from the multiple regression models shed light to the possible reasons for disagreement between self-reported arthritis and reference standard. Recall that the results indicate better SF-36 physical component, and lower disability or WOMAC scores are associated with false-positive; while, obesity, lower PCS, and greater disability or WOMAC scores are associated with false-negative. Previously, Bombard et al. (2005) suggested that individuals with better health have better control of their conditions, making them less likely to report their musculoskeletal signs and symptoms [31]. Conversely, individuals with worse health are more aware of their signs and symptoms and more likely to report them [31]. Thus, women who had arthritis but had otherwise good health were less likely to report any sign and symptom and be classified as false-positive, whereas women who did not have arthritis but had worse overall health were more likely to report their musculoskeletal signs and symptoms and be classified as false-negative.

There are both limitations and strengths to this study. First, our reference standard is not the gold-standard case-definition of arthritis for epidemiological studies. A gold-standard case-definition of arthritis for epidemiological research does not exist [30, 56]. Our choice was based on: a) it allowed us to measure the agreement with clinical signs and symptoms instead of, for example, radiographic evidence which may not link to symptoms or treatment decisions [44]; b) the assessed musculoskeletal signs and symptoms aligned with those recommended in the general practitioner’s guidelines for the diagnosis of arthritis [44]; and c) COPCORD questions have been used to estimate the burden of musculoskeletal conditions in community-living individuals in Australia [40]. Second, our study did not include men. It has been reported that factors associated with the reporting of joint symptoms are different between women and men [57]. Hence, our results might not be generalizable to the male population. However, the present sample was specifically chosen because the research focus of this study was the accuracy of self-reported arthritis in older women. Arthritis is a gendered disease where women have a higher prevalence [1, 3, 5], and more disabilities [3, 9, 10]. This study provides important information about the accuracy of self-reported diagnosed arthritis in a population most affected by arthritis (i.e. older women). Concurrently, our sample represents a strength of this study because: a) survey participants were randomly drawn from an ALSWH cohort which is geographically diverse [36]; and b) the response rate in this study was very high. These factors contribute positively to both the external and internal validity of the findings.

## Conclusion

Self-reported arthritis is one of the most common case-definitions in epidemiological studies. Our results show that the estimated prevalence of arthritis in older community-living women based on self-reported diagnosed arthritis and cases identified by musculoskeletal signs and symptoms were not statistically significantly different. However, our results also show that agreement between self-report diagnosed arthritis and musculoskeletal signs and symptoms was only moderate. Results indicate two-fifths of self-reported arthritis did not have musculoskeletal signs and symptoms in the previous week (false-positives), while one-third of cases identified by signs and symptoms did not report diagnosed arthritis (false-negatives). These findings may suggest that a combined case-definition that includes both reported arthritis and musculoskeletal signs and symptoms be more effectively capture the diagnosed cases that could have fluctuating symptoms as well as the individuals who have the signs and symptoms but have not received a diagnosis; the feasibility and validity of such hybrid case-definition should be examined in future studies. Regression analysis did not find any significant socio-demographic factor or lifestyle factor associated with disagreement once the health variables were included. The results indicate that better general physical health is associated with a false-positive while worse overall health is associated with a false-negative. Researchers who use self-reported diagnosed arthritis as the case-definition should consider these limitations when making interpretation of results and drawing conclusions.

## Additional file

Additional file 1: Table S1. Univariate analysis results for factors associated with false-positive and false-negative. (DOCX 16 kb)

## Abbreviations

ACR: American College of Rheumatology
ALSWH: The Australian Longitudinal Study on Women’s Health
BMI: Body Mass Index
CCQ: Core Questionnaire
CI: Confidence interval
COPCORD: Community Oriented Program for Control of Rheumatic Disease
HAQ: Health Assessment Questionnaire
ILAR: International League against Rheumatism
MCS: The SF-36 mental component summary
MSK: Musculoskeletal
NPV: Negative predictive value
PCS: The SF-36 physical component summary
PPV: Positive predictive value
RACGP: The Royal Australian College of General Practitioners
SD: Standard deviation
SF-36: Short Form 36
WHO: The World Health Organization
WOMAC: Western Ontario and McMaster Universities Osteoarthritis Index
