# Supplementary material for: Discordance between self-reported arthritis and musculoskeletal signs and symptoms in older women
Source: BMC Musculoskelet Disord. 2016 Dec 1;17:494. doi: 10.1186/s12891-016-1349-4 (PMC5133957; doi:10.1186/s12891-016-1349-4)
Supplement: Additional file 1: Table S1. — Univariate analysis results for factors associated with false-positive and false-negative. (DOCX 16 kb) [file 12891_2016_1349_MOESM1_ESM.docx]

### Additional file: Results of univariate analysis

**Table S1** Univariate analysis results for factors associated with false-positive and false-negative

|  | **False-positive** | | |  | **False-negative** | | |
| --- | --- | --- | --- | --- | --- | --- | --- |
| **Characteristics** | **Odds ratio** | **(95% CI)** |  |  | **Odds ratio** | **(95% CI)** |  |
| Age | 0.96 | (0.79-1.16) |  |  | 1.07 | (0.89-1.28) |  |
| Married or *de facto* | 1.24 | (0.65-2.39) |  |  | 0.86 | (0.44-1.71) |  |
| Residing in urban area | 1.20 | (0.68-2.11) |  |  | 0.59 | (0.33-1.05) |  |
| High school or less | 0.68 | (0.39-1.19) | * |  | 0.69 | (0.40-1.19) |  |
| Current smoker | 1.72 | (0.64-4.65) |  |  | 0.96 | (0.35-2.64) |  |
| Obese | 0.69 | (0.38-1.26) | * |  | 1.87 | (1.04-3.35) | * |
| Comorbidity | 0.97 | (0.80-1.17) |  |  | 0.97 | (0.73-1.27) |  |
| PCS | 1.06 | (1.03-1.09) | ** |  | 0.92 | (0.90-0.95) | ** |
| MCS | 1.01 | (0.98-1.03) |  |  | 0.98 | (0.95-1.01) |  |
| HAQ Disability Index | 0.37 | (0.22-0.63) | ** |  | 4.41 | (2.23-8.73) | ** |
| WOMAC total score | 0.97 | (0.95-0.99) | ** |  | 1.05 | (1.02-1.08) | ** |
| **P* ≤ 0.05, ***P* ≤ 0.01. **Abbreviations** CI: confidence interval; PCS: SF-36 physical summary score; MCS: SF-36 mental summary score; HAQ: Health Assessment Questionnaire; WOMAC: Western Ontario and McMaster Universities Osteoarthritis Index. | | | | | | | |
|  |  |  |  |  |  |  |  |
